# Effectiveness of an intervention to overcome influenza vaccine hesitancy in specialty clinic patients

**DOI:** 10.1097/MD.0000000000029786

**Published:** 2022-07-29

**Authors:** Nathaniel J. Webb, Joshua Lindsley, Erica L. Stockbridge, Ashleigh Workman, Conner D. Reynolds, Thaddeus L. Miller, Jean Charles, Michael Carletti, Stefanie Casperson, Stephen Weis

**Affiliations:** a Department of Health Behavior and Health Systems, School of Public Health, University of North Texas Health Science Center, Fort Worth, Texas, United States; b Department of Internal Medicine, Texas College of Osteopathic Medicine, University of North Texas Health Science Center, Fort Worth, Texas, United States; c Department of Dermatology, Medical City Weatherford, Weatherford, Texas, United States; d John Peter Smith Hospital, JPS Health Network, Fort Worth, Texas, United States.

## Abstract

**Background:**

Individuals on immunosuppressive therapies experience greater morbidity and mortality due to vaccine-preventable illnesses, but there are low rates of adherence to immunization guidelines within this population.

**Objective:**

To determine the effectiveness of clinician-led education, patient-centered dialogue, and immediately available immunization on influenza vaccination uptake in patients taking immunosuppressive therapies.

**Method:**

We used a controlled before-and-after quasi-experimental design to evaluate our quality improvement intervention occurring from September 2019 to March 2020, with follow-up through July 2020. The study included 2 dermatology practices wherein nursing staff offered influenza vaccination during patient rooming (standard care). Within each practice, clinicians either implemented the intervention or provided only standard care. Patients received the intervention or standard care depending on the clinician they visited. Patients seen at the 2 clinics during the intervention period were included in analyses if they were taking or newly prescribed immunosuppressant medication at the time of their visit. We examined influenza immunization status for 3 flu seasons: 2017–2018 (preintervention), 2018–2019 (preintervention), and 2019–2020 (intervention).

**Intervention:**

Immunosuppressed patients initially declining an influenza vaccine were provided dermatologist-led education on the benefits of immunization. Dermatologists explored and addressed individual patients’ immunization concerns. Influenza vaccination was then offered immediately postdialogue.

**Results:**

Analyses included 201 dermatology patients who were prescribed or currently taking immunosuppressive medication (intervention group [72.6%], comparison group [27.4%]). During the intervention period, 91.1% of the intervention group received influenza vaccination compared to 56.4% of the comparison group. Vaccination trends from 2018–2019 (preintervention) to 2019–2020 (intervention) differed significantly between groups (χ^2^ = 22.92, *P* < .001), with greater improvement in the intervention group. In 2019–2020, influenza vaccination was more likely in the intervention group relative to the comparison group (odds ratio: 16.22, 95% confidence interval: 5.55–47.38). In the subset of patients that had never received an influenza vaccine, influenza immunization in 2019–2020 was more common in the intervention group (75.8%, 25/33) relative to the comparison group (13.3%, 2/15, *P* < .001).

**Conclusion:**

The intervention successfully addressed vaccine hesitancy and improved influenza immunization rates in an immunosuppressed population receiving care from a specialty clinic. Implementing a similar model across specialty clinics may improve vaccination rates for influenza, coronavirus disease 2019, and other vaccine-preventable illnesses in other populations.

## 1. Introduction

Seasonal influenza is a common, vaccine-preventable respiratory illness,^[1]^ responsible for substantial health care spending, hospitalizations, morbidity, and mortality.^[2–7]^ The Global Influenza Mortality Project estimated the annual influenza-associated respiratory mortality rate of 5.9 deaths per 100,000 population.^[6]^ Persons on immunosuppressive therapy experience greater morbidity and mortality from vaccine-preventable illnesses such as influenza.^[8–15]^ Clinical practice guidelines worldwide recommend routinely vaccinating immunosuppressed patients to reduce complications.^[16,17]^ However, the multinational study on Comorbidities in Rheumatoid Arthritis found only 25.3% of patients on immunosuppressive therapy were receiving influenza vaccinations.^[18]^ Thus, low adherence to immunization guidelines may contribute to influenza complications observed in this population.

An important contributor to patients’ failure to adhere to immunization guidelines is vaccine hesitancy. Vaccine hesitancy is the reluctance or refusal to vaccinate despite the availability of vaccines.^[19]^ In 2019, the World Health Organization identified vaccine hesitancy as 1 of 10 leading threats to global health.^[19]^ Survey data from the US indicates that patients who had not received the influenza vaccine in the previous 5 seasons had greater vaccine hesitancy.^[20]^ Immunosuppressed patients cite concerns of efficacy or safety^[21]^ and time constraints^[21]^ when describing reasons for nonvaccination. Unvaccinated patients often report that their physician did not offer vaccines to them.^[21,22]^ Affordability of vaccinations^[23]^ and lack of insurance^[22]^ are additional barriers to vaccine uptake. Together, these factors lead to low adherence to influenza immunization guidelines.

To improve influenza immunization rates, clinicians and health systems must implement strategies that improve convenience, increase patient confidence, and overcome vaccine hesitancy. Practice-proven strategies to increase influenza vaccination rates include clinicians making strong recommendations,^[24–28]^ broadening the vaccination window,^[29]^ providing vaccinations in nontraditional settings,^[30–33]^ and utilizing standing orders.^[34–36]^ One previous 10-year study by the Veterans Administration Medical Center in Minneapolis, Minnesota, found that combining standing orders, physician delivered education, walk-in clinic options, and the use of standardized, preprinted documentation forms increased influenza vaccinations by 58% to 84%.^[37]^

Providing vaccinations in nontraditional settings is an underused strategy to increase vaccination rates. Historically, vaccine delivery occurred in ambulatory primary care settings. However, in recent years a substantial number of adults have received vaccinations in other settings. According to a Centers for Disease Control and Prevention survey, the most common locations for influenza vaccination in adults are physicians’ offices (39%), pharmacies/stores (22%), and workplaces (16%).^[38]^ Providing additional vaccination opportunities in nonprimary care settings would likely improve access and vaccination uptake in persons who would otherwise go unvaccinated.^[32]^ However, there is little research investigating specialty clinics offering influenza immunization to immunosuppressed patients.

Our specialty clinic observed incomplete influenza immunizations among patients receiving immunosuppressive medications. Following Advisory Committee on Immunization Practices recommendations, we designed a quality improvement (QI) project implementing a multifactorial approach centered around clinician-led recommendations and same-day influenza vaccination availability in specialty clinics. We evaluated the results of the QI project and presented our findings here.

## 2. Methods

This project was approved as exempt category research by the North Texas Institutional Review Board.

### 2.1. Study design and participants

We used a before-and-after quasi-experimental study design to quantify the effectiveness of the QI project on increasing influenza vaccinations for immunosuppressed dermatology patients. Dermatology patients seen at 2 practices were eligible for inclusion in analyses if they were taking or newly prescribed autoimmune suppressant medication at the time of their visit. Immunosuppressant therapies included biologics, antimetabolites, oral corticosteroids, and other immunosuppressive medications.

Within each practice, clinicians either implemented the intervention or provided standard care. Thus, patients received the intervention or standard care depending on the clinician they visited. The QI project took place between September (practice #1) or November (practice #2) of 2019 through March 2020. Due to the nature of the intervention and assignment method, clinicians were not blinded to patients’ study condition assignments. All eligible patients visiting either practice during this period were included in analyses. Participants were followed for flu immunization through July 2020.

Both clinics are in Tarrant County, Texas, which had a population of 2,102,515 in 2019. The demographic makeup of Tarrant County during 2019 was 45.3% White, 29.5% Hispanic, 17.9% Black, 5.8% Asian, and 3.8% other.^[39]^

### 2.2. Standard care

Prior to and during the period of the QI intervention, the influenza vaccine was on the formulary of both clinics. There were no system differences between the clinic locations in vaccine administration, administrative and medical staff, and electronic medical records documentation. All dermatology clinic patients received vaccine information statements produced by the Centers for Disease Control and Prevention for influenza as part of front desk check-in processes. Nursing staff then offered influenza vaccination during patient rooming through a standard delegated order.

### 2.3. Quality improvement intervention

Following the receipt of standard care, a dermatologist educated immunization-declining patients about immunization. Education included verbally reminding them of the increased risk of infection associated with immunosuppressive medications. We then explained the risk-reduction benefits of immunization. Patients who were still uncertain were then directly asked about their reasons for their hesitancy. Those reasons were then addressed.^[40]^ Postdiscussion, the dermatologist immediately offered the flu vaccine to patients who had not already received a seasonal immunization. Immunizations were provided during the same office visit.

### 2.4. Data source and measures

Data for demographic, patient health, and most health care delivery variables were retrospectively collected through electronic medical record. Additional information about influenza immunization history was obtained through postvisit telephone calls for the comparison group and a patient survey administered during the visit for the QI group (see Survey, Supplemental Digital Content, which is a copy of the patient survey, http://links.lww.com/MD/G815). In that survey, patients also provided information about reasons for not previously receiving influenza vaccination. Reasons included “Fear of future adverse reaction,” “History of adverse reaction,” “Did not receive a recommendation by clinician to get the vaccine,” “Did not know what the vaccine was for,” “Forgot to get the vaccine,” and “Other.” For analysis, the “Did not know…,” “Forgot …,” and “Other” responses were collapsed into one “Other” category. The “History…” response was excluded because patients without prior flu vaccinations reported no prior adverse reactions to flu vaccinations. Data from the clinics were combined, and a group variable indicated if a patient visited a QI or comparison (i.e., standard care) clinician.

We determined each patient’s flu immunization status for the 2017–2018 (preintervention), 2018–2019 (preintervention), and 2019–2020 (QI intervention) flu seasons based on immunizations occurring July 1st through June 30th of each immunization period.^[41]^ For the 2019–2020 flu season, the patient was categorized as receiving the flu vaccine previsit, at the visit, postvisit, or never.

Demographic, care delivery, and health status variables were also included. Demographic variables included age, race/ethnicity, and sex. Care delivery variables included insurance status and past year office-based health care visits to any clinician. Health status variables included the patient’s autoimmune disease, number and type of immunosuppressant medications, and number of comorbidities. Comorbidities included asthma, coronary artery disease, cancer, chronic pain, chronic obstructive pulmonary disease, chronic renal failure, diabetes, high cholesterol, hypertension, hypothyroidism, psychiatric disorder, seizures, gastroesophageal reflux disease, or osteoarthritis.

### 2.5. Statistical analyses

All analyses were conducted at the individual patient level. To confirm the comparability of QI and comparison groups, we tested differences in demographics, care delivery, health status, and past flu vaccination using chi-square tests, Fischer exact tests, or Wilcoxon rank-sum tests to estimate significance, as appropriate. We then examined vaccinations occurring during the 2019–2020 flu season to estimate the impact of the intervention. Using chi-square tests, we compared QI and comparison patients’ receipt of a flu vaccine during that season. Next, we examined group differences in having received the flu vaccination prior to the visit, at the visit, after the visit, or never. We examined adjusted differences in flu vaccination for the QI/comparison groups, controlling for gender, age, race/ethnicity, insurance, previous care contacts, type of visit, and past immunosuppressive use.

To identify whether vaccination rates changed over time and if changes differed by group, we conducted difference-in-difference modeling comparing flu vaccination status during the 2017–2018, 2018–19, and 2019–20 flu seasons. Unadjusted analyses compared flu vaccination status of the groups during different seasons through logistic regression, modeling flu season, study group, and the season by study group interaction. Adjusted analyses expanded the previous model by controlling for gender, age, insurance, previous care contacts, and visit type. These analyses were used to estimate unadjusted and adjusted predicted probabilities of flu vaccination for each flu season for both groups. For interpretation, we conducted contrast analyses examining overall interaction significance, the simple effect of group by season, and reverse adjacent interaction significance.

Our final, secondary analyses included persons who had never received a flu vaccination prior to the 2019–2020 season. Within this subset, we examined differences between QI/comparison groups in the likelihood of receiving a 2019–2020 flu vaccination. We analyzed data for patients in the QI group with no prior vaccine to identify associations between persistent nonacceptance and the 2 most common patient-reported reasons for not receiving prior influenza vaccines: fear of adverse reaction and having had no previous recommendation. Fisher exact tests were used for all subset analyses.

Data were managed in Microsoft Access and analyses were conducted in Stata SE V.16.1 (StataCorp, College Station, TX). All statistical testings were 2-sided with significance tested at a *P* value of <.05.

## 3. Results

A total of 201 patients met inclusion criteria (QI = 146, 72.64%; comparison = 55, 27.36%); no patients were lost to follow-up. Except for the comparison group having a higher proportion of new patients (34.55%) compared to the QI group (19.18%, *P* = .02), we observed no significant difference between the groups in demographic, care delivery, health care, and flu immunization history variables (*P* > .05 for each; Table 1). For patients who had never had a flu vaccine prior to the 2019–2020 season, the reasons given for nonreceipt did not significantly vary between QI and comparison groups (*P* > .05 for each; Table 1).

**Table 1:** Sample demographics, health status, and self-reported reason for prior vaccination nonreceipt, in total and by QI versus comparison group.

Variable	Total	Comparison group	QI group	*P* value
	N = 201, n (%)	N = 55, n (%)	N = 146, n (%)	
Gender				
Female	132 (66.44)	35 (63.64)	97 (66.44)	
Male	69 (34.33)	20 (36.36)	49 (33.56)	.71
Age, yr				
≤34	36 (17.91)	7 (12.73)	29 (19.86)	
35–44	36 (17.91)	7 (12.73)	29 (19.86)	
45–54	42 (20.90)	16 (29.09)	26 (17.81)	
55–64	51 (25.37)	15 (27.27)	36 (24.66)	
≥65	36 (17.91)	16 (29.09)	26 (17.81)	.31
Race/ethnicity				
Non-Hispanic White	76 (37.81)	17 (30.91)	59 (40.41)	
Non-Hispanic Black	48 (23.88)	19 (34.55)	29 (19.86)	
Hispanic	64 (31.84)	15 (27.27)	49 (33.56)	
Other	13 (6.47)	4 (7.27)	9 (6.16)	.16
Insurance				
Private	22 (10.95)	5 (9.09)	17 (11.64)	
Public	159 (79.1)	43 (78.18)	116 (79.45)	
Uninsured	20 (9.95)	7 (12.73)	13 (8.9)	.66
Previous care contact				
0–5	78 (38.81)	17 (30.91)	61 (41.78)	
6–12	76 (37.81)	20 (36.36)	56 (38.36)	
≥13	47 (23.38)	18 (32.73)	29 (19.86)	.13
Visit type				
Initial	47 (23.38)	19 (34.55)	28 (19.18)	
Follow-up	154 (76.62)	36 (65.45)	118 (80.82)	.02
Total comorbidities				
None	39 (19.4)	9 (16.36)	30 (20.55)	
1–3 conditions	112 (55.72)	32 (58.18)	80 (54.79)	
≥4 conditions	50 (24.88)	14 (24.45)	36 (24.66)	.46
Past immunosuppressives				
No	34 (16.92)	10(18.18)	24 (16.44)	
Yes	167 (83.08)	45 (81.82)	122 (83.56)	.77
Autoimmune disease				
Psoriasis with or without psoriatic arthritis	141 (70.15)	40 (72.73)	101 (69.18)	
Hidradenitis suppurativa	19 (9.45)	5 (9.09)	14 (9.59)	
Connective tissue disease	17 (8.46)	3 (5.45)	14 (9.59)	
Rheumatoid arthritis	11 (5.47)	4 (7.27)	7 (4.79)	
Vesiculobullous disease	8 (3.98)	1 (1.82)	7 (4.79)	
Atopic dermatitis	2 (1)	1 (1.82)	1 (0.68)	
Inflammatory bowel disease	1 (0.5)	0	1 (0.68)	
Chronic lichenoid inflammatory disease	1 (0.5)	0	1 (0.68)	
Organ transplant	1 (0.5)	1 (1.82)	0	.66
Current number of immunosuppressive medications prescribed				
1	162 (80.6)	45 (81.82)	117 (80.14)	
2	35 (17.41)	9 (16.36)	26 (17.81)	
3	4 (1.99)	1 (1.82)	3 (2.05)	.79
Biologics				
No	40 (19.9)	14 (25.45)	26 (17.81)	
Yes	161 (80.1)	41 (74.55)	120 (82.19)	.23
Other drugs				
No	138 (68.66)	37 (67.27)	101 (69.18)	
Yes	63 (31.34)	18 (32.73)	45 (30.82)	.80
Ever had a past influenza vaccine				
No	48 (23.88)	15 (27.27)	33 (22.60)	
Yes	153 (76.12)	40 (72.73)	113 (77.40)	.49
Flu season – received influenza immunization				
2017–2018	90 (44.78)	23 (41.82)	67 (45.89)	.61
2018–2019	97 (48.26)	25 (45.45)	72 (49.32)	.63
2019–2020	164 (81.59)	31 (56.36)	133 (91.1)	<.001
Reason for no previous influenza vaccine*				
Fear of adverse reactions				
No	34 (70.83)	11 (69.70)	23 (69.70)	
Yes	14 (29.17)	4 (26.67)	10 (30.30)	.80
No previous recommendation				
No	19 (39.58)	7 (46.67)	12 (36.36)	
Yes	29 (60.42)	8 (53.33)	21 (63.64)	.50
Skipped question				
No	45 (93.75)	13 (86.67)	32 (96.97)	
Yes	3 (6.25)	2 (13.33)	1 (3.03)	.23
Other				
No	42 (87.50)	14 (93.33)	28 (84.85)	
Yes	6 (12.50)	1 (6.67)	5 (15.15)	.65

In unadjusted and adjusted analyses of 2019–2020 flu season data, we found that persons in the QI group were significantly more likely than those in the comparison group to receive a flu vaccination (unadjusted *P* < .001; Table 2). Specifically, 91.10% of patients in the QI group received a flu vaccination, while 56.36% of patients in the comparison group received a flu vaccination during the 2019–2020 season (Table 2). Further, the QI group had significantly higher adjusted odds of receiving a flu vaccine in 2019–2020 (adjusted odds ratio [OR]: 16.22, 95% confidence interval [CI]: 5.55–47.38, *P* < .001; Table 3). The groups also differed significantly in when patients received a 2019–2020 flu vaccination (*P* < .001; Table 2). Most notably, in unadjusted analyses, 45.89% of the QI group received a flu vaccination at the visit to the dermatology clinic compared to 0% of the comparison group (Table 2).

**Table 2:** Unadjusted comparison of QI vs comparison groups for receiving influenza vaccine during 2019–2020 flu season, in total and by timing of immunization (n = 201).

Group	Received flu vaccine in 2019–2020 season (total)	*P* value	Received 2019–2020 flu vaccine prior to visit n (%)	Received 2019–2020 flu vaccine at visit n (%)	Received 2019–2020 flu vaccine after visit n (%)	Never received 2019–2020 flu vaccine n (%)	*P* value
	n (%)						
QI group (n = 146)	133 (91.1)	<.001	61 (41.78)	67 (45.89)	5 (3.42)	13 (8.9)	<.001
Comparison group (n = 55)	31 (56.36)		25 (45.45)	0 (0)	6 (10.91)	24 (43.64)	
Combined group	164 (81.59)		86 (42.79)	67 (33.33)	11 (5.47)	37 (18.41)	

**Table 3:** Results of multivariable logistic regression model examining flu immunization status during the 2019–2020 flu season (n = 201).

Variable	Category	OR (95% CI)	*P* value	Avg. predicted probability (95% CI)
Group	Comparison	1.00 (ref)		0.55 (0.43, 0.67)
	QI	16.22 (5.55–47.38)	<.001	0.91 (0.87–0.95)
Gender	F	1.00 (ref)		0.80 (0.74–0.85)
	M	1.76 (0.67–4.61)	.25	0.85 (0.78–0.92)
Age, yr	≤44	1.00 (ref)		0.71 (0.62–0.80)
	45–64	4.32 (1.40–13.30)	.01	0.86 (0.81–0.92)
	≥65	6.41 (1.26–32.50)	.03	0.89 (0.80–0.99)
Insurance	Private	1.00 (ref)		0.89 (0.77–1.01)
	Public	0.64 (0.11–3.55)	.61	0.85 (0.80–0.90)
	Uninsured	0.06 (0.01–0.44)	.006	0.52 (0.35–0.70)
Office-based care contacts in prior 12 mo	0–5	1.00 (ref)		0.80 (0.73–0.88)
	6–12	1.06 (0.35–3.22)	.92	0.81 (0.73–0.89)
	≥13	1.55 (0.46–5.28)	.48	0.84 (0.76–0.93)
Type of visit	Initial	1.00 (ref)		0.75 (0.65–0.85)
	Follow-up	2.42 (0.91–5.86)	.08	0.84 (0.79–0.89)
Past immunosuppressives?	No	1.00 (ref)		0.82 (0.71–0.92)
	Yes	0.99 (0.30–3.24)	.11	0.82 (0.77–0.87)

Adjusted analyses of 2019–2020 flu season data also found significant associations between flu vaccination and 2 covariates. Relative to the under 44 years age group, patients in age groups 45 to 64 years (OR: 4.32, 95% CI: 1.40–13.30, *P*= .01) and 65 years and older (OR: 6.41, 95% CI: 1.26–32.50, *P* = .03) had higher adjusted odds of being vaccinated (Table 3). Finally, uninsured patients compared to privately insured patients had significantly lower adjusted odds of being vaccinated (OR: 0.06, 95% CI: 0.01–0.44, *P* = .006; Table 3).

**Figure 1.**
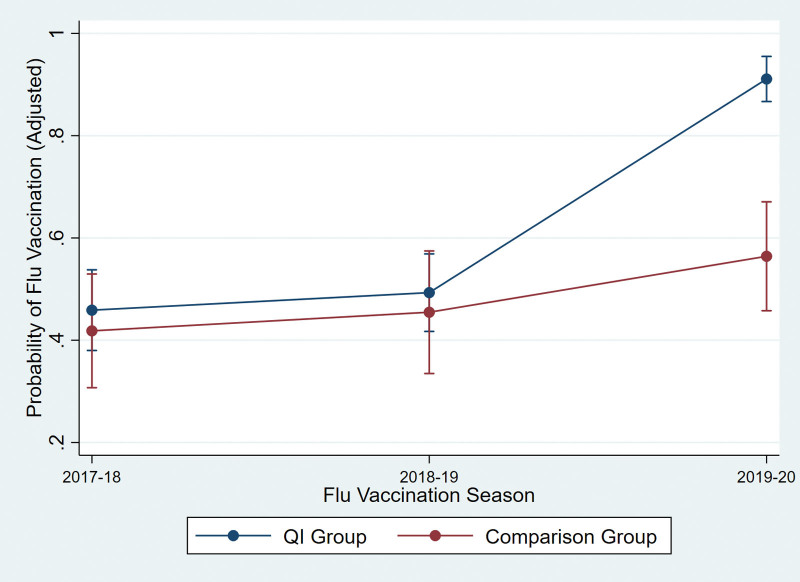
Adjusted predicted probability of receiving flu vaccine by QI and comparison group through 3 flu seasons, 2017–2018, 2018–2019, 2019–2020. QI = quality improvement.

Our difference-in-difference models identified significant overall interactions between season and group (*P* < .001, both unadjusted and adjusted), indicating that changes in flu vaccination over time differed for the QI and intervention groups (Fig. 1). Specifically, vaccination trends from 2018–2019 to 2019–2020 differed significantly between groups, with greater improvement observed in the QI group (*P* < .001, both unadjusted and adjusted). Conversely, changes from 2017–2018 to 2018–2019 did not significantly differ for the 2 groups (unadjusted *P* = .97; adjusted *P* = .97). In 2019–2020, vaccination was significantly more likely in the QI group relative to the comparison group (*P* < .001, both unadjusted and adjusted); however, the groups did not differ significantly during 2017–2018 (unadjusted *P* = .61; adjusted *P* = .56) or 2018–2019 (unadjusted *P* = .63; adjusted *P* = .60). This pattern suggests that the QI group’s greater increase in vaccination uptake relative to the comparison group likely resulted from the QI intervention during 2019–2020 rather than being attributable to differing ongoing trends. Logistic model output and contrast analyses are also provided (see eTable 1, Supplemental Digital Content, http://links.lww.com/MD/G815, which contains the logistic model output, and eTable 2, Supplemental Digital Content, http://links.lww.com/MD/G815, which contains the results of the contrast analyses). Probabilities generated by the 2 models are detailed in Table 4, and adjusted probabilities are depicted in Figure 1.

**Table 4:** Percentage* of individuals receiving influenza immunization, by QI and comparison group for 3 flu seasons (n = 201).

Group	Unadjusted percentages (95% CI)			*P* value	Adjusted percentages (95% CI)			*P* value
	**2017–2018**	**2018–2019**	**2019–2020**		**2017–2018**	**2018–2019**	**2019–2020**	
QI group (N = 146)	45.9 (37.8–54.0)	49.3 (41.2–57.4)	91.1 (86.5–95.7)	<.001	45.9 (38.0–53.8)	49.3 (41.7–56.9)	91.1 (86.7–95.5)	<.001
Comparison group (N = 55)	41.8 (28.7–54.9)	45.5 (32.3–58.6)	56.4 (43.2–69.5)		41.8 (30.7–53.0)	45.5 (33.5–57.5)	56.4 (45.8–67.1)	

The adjusted difference-in-difference model also yielded significant age differences in immunizations. Persons in the 45 years and older age categories were more likely to be immunized relative to those 44 years and younger (*P* < .001 for both; eTable 1, Supplemental Digital Content, http://links.lww.com/MD/G815). Further, uninsured individuals were less likely to receive the flu vaccination compared to privately insured patients (*P* = .009; eTable 1, Supplemental Digital Content, http://links.lww.com/MD/G815). Finally, patients visiting for a follow-up dermatology visit had higher odds of receiving a flu vaccination relative to new patients (*P* = .03; eTable 1, Supplemental Digital Content, http://links.lww.com/MD/G815).

Vaccine uptake within the 48 patients who had never received a flu vaccination before the 2019–2020 season differed for the QI and comparison groups (*P* < .001). Relative to the comparison group, first-time vaccinations in 2019–2020 were significantly more common in the QI group (QI: 75.76%; 25/33 vs comparison: 13.33%; 2/15). Further, of the 33 patients in the QI group who had never previously been vaccinated, there was a significant association between receipt of a flu vaccine in 2019–2020 and the self-reported reasons for not having a previous flu vaccine. However, the directions of the associations differed. Previously nonvaccinated persons in the QI group who said they had received no recommendation in the past were significantly more likely to receive a vaccination in 2019–2020 (90.48%, 19/21) than those not reporting that reason (50.00%, 6/12; *P* = .02). Conversely, those who said they had not previously been vaccinated because they feared adverse reactions were less likely to receive a vaccination (50.00%, 5/10) than those not reporting that reason (86.96%, 20/23; *P* = .04).

## 4. Discussion

Clinical guidelines recommend routinely vaccinating immunosuppressed patients to reduce hospitalizations, morbidity, and mortality due to preventable illnesses, such as influenza.^[16,17]^ However, this recommendation is poorly implemented worldwide, leaving many patients at increased risk.^[18,42–45]^ Our QI project was designed to address disparities in influenza vaccination among such patients.^[40]^ The intervention was associated with a 98% increase in the probability of influenza vaccine uptake (45.9%–91.1%). These data demonstrate the efficacy of patient-specific education and convenient onsite vaccinations at alternate clinical locations to improve vaccine coverage among patients receiving immunosuppressive therapy.

Multiple interrelated factors lead to vaccine hesitancy, including historical, political, sociocultural, interpersonal, and individual beliefs.^[19,46,47]^ Mistrust in governmental and pharmaceutical institutions involved in vaccine creation and distribution fuel vaccine hesitancy.^[23,48]^ Conversely, patients typically trust clinicians to act in their best interests, especially when clinicians exhibit interpersonal competence. Previous literature cites the lack of clinician recommendation as the most common reason for low vaccine uptake among immunosuppressed patients.^[21,22,49,50]^ Our data illustrate that by building and leveraging patients’ trust in clinicians, addressing individual patients’ immunization concerns,^[40,51]^ and providing a clear recommendation, patients’ vaccine hesitancy can be overcome.^[40]^ Of QI participants who had never received an influenza vaccination nor recalled recommendations to do so, 63.64% (21/33) accepted influenza vaccination.

Our intervention involved person-centered 2-way communication about vaccination concerns and experiences.^[40]^ While compulsory vaccine mandates have been widely effective at increasing vaccination coverage,^[52–54]^ mandates may result in increased vaccine exemption-seeking behavior.^[55,56]^ Notably, compulsory vaccination requirements may reduce voluntary vaccinations in individuals with negative attitudes regarding vaccines. In our study, 50% of patients in the QI group reporting fear of adverse reactions were vaccinated for the first time during the intervention period. This study shows a patient-centered approach can successfully address vaccine hesitancy. Further, organizational support and coordination across the health care team provided a conducive environment for addressing vaccine hesitancy.

Despite the presence of our intervention, lack of insurance remained a barrier to vaccine uptake. Cost barriers to influenza immunization disproportionally affect patients without health insurance.^[57,58]^ A 2012 National Health Interview Survey exemplified this disparity, showing uninsured adults’ influenza coverage was 14.4% compared to 44.3% in the insured adult population.^[59]^ We observed a similar finding in our QI project. Uninsured patients were the only group where the QI intervention was not successful (adjusted *P* = .006; Table 4). Conversely, patients enrolled in the county health insurance program (JPS Connection) were willing to accept the QI intervention. Similar public insurance programs implemented elsewhere could reduce vaccine disparity and increase uninsured patients’ opportunities to be immunized.

Importantly, our intervention was successful in overcoming vaccine hesitancy in a vulnerable population. This success was rooted in addressing important influences on health decision-making and is likely generalizable to the current coronavirus disease 2019 (COVID-19) vaccine. These findings are timely, as a recent Pew Research survey found that 3 in 10 individuals in the United States currently do not plan to receive the vaccine for COVID-19.^[60]^ These individuals cited fear of adverse reactions, concerns about vaccine development processes, and need for more information as primary reasons for their COVID-19 vaccine hesitancy.^[40,60]^ Our evidence-based QI intervention shows that vaccine hesitancy can be overcome using a multifactorial strategy involving organizational commitment, clinician patient-centered education, addressing the individual patient’s immunization concerns, and same-day vaccination. Ensuring appropriate COVID-19 vaccine uptake among potentially reluctant immunosuppressed patients is of major importance. Our QI intervention suggests an accessible and effective means to do so. We saw a 12.5-fold increase among individuals with no history of accepting influenza vaccine, suggesting our intervention may provide important motivations for especially hesitant patients.

Our study had various limitations. Due to the small sample size of our data, we were unable to analyze the site-specific differences between the 2 clinic locations,^[61]^ nor could we examine racial or ethnic differences.^[62]^ Similarly, our data is specific to the north Texas area where our clinics served primarily low socioeconomic level patients. These factors limit the study’s ability to be generalizable to the US population. Additionally, the retrospective data collection may have resulted in missing information. As a result, future studies should include larger sample sizes and greater geographic and socioeconomic level variability. Finally, we focused on acceptance of inactivated injectable flu vaccines, so additional research is needed to determine if acceptance in the presence of the intervention would have varied by vaccine type (e.g., live-attenuated, toxoid). Despite these limitations, our study clearly shows the impact of strong clinician recommendations on improving vaccine uptake in immunosuppressed individuals.

This study successfully addressed vaccine hesitancy and improved immunization rates related to influenza in an adult, immunosuppressed population receiving care from a specialty clinic. Through direct patient education, onsite vaccinations, and organizational buy-in, we vaccinated 91.1% of our study population, higher than the 70% national target set by Healthy People 2020.^[63]^ Implementing a similar model across specialty clinics may prove valuable for improving vaccination rates for influenza, COVID-19, and other vaccine-preventable illnesses in immunosuppressed populations.

### Author contributions

Conceptualization: Joshua Lindsley, DO; Ashleigh Workman, DO; Jean Charles, DO; Michael Carletti, DO; Stephen Weis, DO

Data curation: Nathaniel J. Webb, MPH; Joshua Lindsley, DO; Erica L. Stockbridge, PhD

Formal analysis: Nathaniel J. Webb, MPH; Erica L. Stockbridge, PhD

Investigation: Joshua Lindsley, DO; Ashleigh Workman, DO; Jean Charles, DO; Michael Carletti, DO; Stefanie Casperson RN, BSN; Stephen Weis, DO

Methodology: Nathaniel J. Webb, MPH; Erica L. Stockbridge, PhD; Thaddeus L. Miller, DrPH, MPH; Michael Carletti, DO; Stephen Weis, DO

Project administration: Joshua Lindsley, DO; Erica L. Stockbridge, PhD; Thaddeus L. Miller, DrPH, MPH; Michael Carletti, DO; Stephen Weis, DO

Resources: Stephen Weis, DO

Software: Nathaniel J. Webb, MPH; Erica L. Stockbridge, PhD

Supervision: Stephen Weis, DO

Validation: Nathaniel J. Webb, MPH; Erica L. Stockbridge, PhD

Visualization: Erica L. Stockbridge, PhD

Writing – original draft: Nathaniel J. Webb, MPH; Joshua Lindsley, DO; Conner D. Reynolds, DO, MS, CPPS; Stephen Weis, DO

Writing – review & editing: Nathaniel J. Webb, MPH; Erica L. Stockbridge, PhD; Ashleigh Workman, DO; Thaddeus L. Miller, DrPH, Jean Charles, DO; Michael Carletti, DO; Stefanie Casperson RN, BSN; Stephen Weis, DO

## Supplementary Material



## Footnotes

* Includes only the subset of individuals without a previous flu vaccine.

* Percentages are the average predicted probabilities calculated based on results of unadjusted and adjusted logit models (see eTable 1, Supplemental Digital Content, http://links.lww.com/MD/G815); probabilities multiplied by 100 and expressed as percentages.
