# Supplementary material for: Effectiveness of an intervention to overcome influenza vaccine hesitancy in specialty clinic patients
Source: Medicine (Baltimore). 2022 Jul 29;101(30):e29786. doi: 10.1097/MD.0000000000029786 (PMC9333474; doi:10.1097/MD.0000000000029786)
Supplement: Supplementary file 1 [file medi-101-e29786-s001.pdf]

Questionnaire

<u>Demographics:</u>

1) Gender: M or F

2) Race (circle one): African American Caucasian Hispanic or Latino Asian American Indian or Alaskan native Native Hawaiian or Other Pacific Islander White

3) Skin disease: ____________________

4) Please list immunosuppressive medications and also include how long you have been on each:
   a.
   b.
   c.
   d.

5) Who is prescribing your immunosuppressive medications?
   a. UNT Dermatology
   b. Other Dermatologist
   c. Rheumatologist
   d. Other

<u>Prior Immunization history:</u>

6) Have you ever received the influenza vaccine?
   a. Yes
   b. No

7) If yes, when?
   a. 2018
   b. 2017
   c. 2016
   d. Other:

8) If no, why not? (choose all that apply)
   a. History of adverse reaction
   b. Fear of future adverse reaction
   c. Did not receive a recommendation by clinician to get the vaccine
   d. Did not know what the vaccine was for
   e. Forgot to get the vaccine
   f. Other

9) Have you ever received the pneumococcal vaccine?
    a. Yes
    b. No

10) If yes, when?
    a. Please list the year(s) received:

11) If no, why not? (choose all that apply)
    a. History of adverse reaction
    b. Fear of future adverse reaction
    c. Did not receive a recommendation by clinician to get the vaccine
    d. Did not know what the vaccine was for
    e. Forgot to get the vaccine
    f. Other

Current Immunizations:

12) Vaccinations recommended today:
    a. Influenza
    b. PCV13
    c. PPSV23

13) Will you be receiving your vaccine today?
    a. Yes
    b. No

14) If no, why not? (choose all that apply)
    a. History of adverse reaction
    b. Fear of future adverse reaction
    c. Did not receive a recommendation by clinician to get the vaccine
    d. Did not know what the vaccine was for
    e. Forgot to get the vaccine
    f. Other

15) Attitude towards vaccines in general
    a. Favorable
    b. Not favorable
    c. No opinion

eTable 1: Results of unadjusted and adjusted (multivariable) logistic regression models examining influenza immunization by season, group, season by group, and other descriptive characteristics.

		Unadjusted		Adjusted	
**Variable**	**Category**	**OR (95% CI)**	**p-value**	**OR (95% CI)**	**p-value**
Flu Season	2017-18	1.00 (ref)		1.00 (ref)	
	2018-19	1.15 (0.83, 1.58)	0.40	1.18 (0.81, 1.71)	0.40
	2019-20	12.06 (6.82, 21.34)	<0.001	18.96 (9.55, 37.66)	<0.001
Group	QI	1.00 (ref)		1.00 (ref)	
	Comparison	0.85 (0.45, 1.59)	0.61	0.82 (0.43, 1.58)	0.56
Season*Group	2017-18*QI	1.00 (ref)		1.00 (ref)	
	2017-18*Comp	1.00 (ref)		1.00 (ref)	
	2018-19 QI	1.00 (ref)		1.00 (ref)	
	2018-19*Comp	1.01 (0.60, 1.70)	0.97	1.01 (0.55, 1.86)	0.97
	2019-20*QI	1.00 (ref)		1.00 (ref)	
	2019-20*Comp	0.15 (0.07, 0.31)	<0.001	0.11 (0.05, 0.25)	<0.001
Sex	Female	n/a	n/a	1.00 (ref)	
	Male	n/a	n/a	1.06 (0.63, 1.78)	0.83
Age	<=44	n/a	n/a	1.00 (ref)	
	45-64	n/a	n/a	3.00 (1.69, 5.33)	<0.001
	>=65	n/a	n/a	3.60 (1.79, 7.25)	<0.001
Insurance	Private	n/a	n/a	1.00 (ref)	
	Public	n/a	n/a	1.34 (0.65, 2.75)	0.43
	Uninsured	n/a	n/a	0.22 (0.07, 0.68)	0.01
Count of past year office-based contacts	0-5	n/a	n/a	1.00 (ref)	
	6-12	n/a	n/a	1.24 (0.70, 2.17)	0.46
	>=13	n/a	n/a	1.16 (0.60, 2.24)	0.66
Visit Type	Initial	n/a	n/a	1.00 (ref)	
	Follow-up	n/a	n/a	2.13 (1.08, 4.19)	0.03

eTable 2: Results of contrast analyses[a] identifying significant differences in influenza immunization by season and group.

		Unadjusted		Adjusted	
		Chi-Squared	p-value	Chi-Squared	p-value
Overall interaction effect		28.68	<0.001	28.57	<0.001
Season by season interaction effects	2018-19 versus 2017-18	0.00	0.97	0.00	0.97
	2019-20 versus 2018-19	22.92	<0.001	23.09	<0.001
Group differences by year	2017-18	0.27	0.61	0.34	0.56
	2018-19	0.24	0.63	0.28	0.60
	2019-20	26.91	<0.001	32.00	<0.001

[a] Based on results of adjusted logistic regression model detailed in eTable 1. Results represent post-estimation contrast analyses.
